# Hypokinetic Hypertrophic Cardiomyopathy: A Rare Case of a Spontaneously Regressive Form in a Newborn

**DOI:** 10.7759/cureus.64186

**Published:** 2024-07-09

**Authors:** Inasse Lamouri, Mohammed ECH-Chebab, Anass Ayyad, Sahar Messaoudi, Abdeladim Babakhouya, Rim Amrani

**Affiliations:** 1 Department of Pediatrics, Faculty of Medicine and Pharmacy, Mohammed VI University Hospital, Mohammed I University, Maternal-Child and Mental Health Research Laboratory, Oujda, MAR; 2 Department of Neonatology and Intensive Care Unit, Faculty of Medicine and Pharmacy, Mohammed VI University Hospital, Mohammed I University, Oujda, MAR; 3 Department of Neonatology and Intensive Care Unit, Faculty of Medicine and Pharmacy, Mohammed VI University Hospital, Mohammed I University, Maternal-Child and Mental Health Research Laboratory, Oujda, MAR; 4 Department of Pediatrics, Department of Pediatric Cardiology, Faculty of Medicine and Pharmacy, Mohammed VI University Hospital, Mohammed I University, Oujda, MAR

**Keywords:** echocardiography, heart failure, hypokinesia, newborn, hypertrophic cardiomyopathy

## Abstract

Hypertrophic cardiomyopathy (HCM) of the newborn is a rare condition, characterized by great clinical variability, with a relative paucity of data on the pediatric population, especially newborns. Early diagnosis can have an impact on the patient's life course and prevent progression to sudden death. In this article, we report the case of a newborn admitted with late-onset neonatal respiratory distress, complicated by heart failure. The newborn was matured by two antenatal injections of betamethasone, which were received as part of a threat of premature delivery. Echocardiography revealed hypokinetic HCM. The rapidity of the establishment of the diagnosis contributed to the patient's survival and improvement within a few weeks under well-managed medical treatment. A complete workup was conducted, with negative results. The most suggested explanation for this condition was the use of antenatal corticosteroids.

## Introduction

According to the World Health Organization, cardiomyopathies are defined as a group of myocardial diseases characterized by mechanical and/or electrical dysfunction, resulting in ventricular hypertrophy or dilation [1,2]. Hypertrophic cardiomyopathy (HCM) is an exceptional entity characterized by left ventricular hypertrophy without dilation of cardiac chambers, typically asymmetric and predominantly affecting the interventricular septum. Occasionally, the right ventricle may also be involved [2-4]. The National Diagnostic and Care Protocol (PNDS) defines HCM by measuring the maximal end-diastolic thickness of the left ventricular wall, interpreted based on the child's age and body surface area, with a Z-score ≥ 2 standard deviations considered significant. Approximately 25% of cases present with a left intraventricular pressure gradient, termed hypokinetic when muscle contractility is decreased. Neonatal onset of HCM remains rare and exhibits a variety of mechanisms and etiologies [5]. Often familial in origin, this condition involves genes encoding sarcomere proteins [1,4]. Diagnostic or management delays can lead to severe complications, including sudden death, necessitating appropriate management [1]. This case report describes a newborn admitted with heart failure, diagnosed with hypokinetic HCM, and illustrates a favorable outcome. We discuss the diagnostic, therapeutic, and prognostic challenges associated with this condition in neonates, emphasizing the critical importance of a multidisciplinary approach to their management.

## Case presentation

The newborn was seventeen days old, female, born from a non-consanguineous marriage, from a well-followed full-term pregnancy, delivered vaginally with a birth weight of 3,100 g. There was a positive history of vulvovaginitis during pregnancy, with the onset of threatened preterm labor at 33 weeks, for which the mother received two intramuscular injections of betamethasone 12 mg, repeated at a 24-hour interval. There were no signs of gestational diabetes or maternal thyroid dysfunction. The mother reported noticing fatigue in her newborn during breastfeeding since birth.

The newborn was hospitalized for neonatal respiratory distress with feeding refusal. Clinical examination revealed a slightly pale, tonic, responsive, afebrile newborn with a weak sucking reflex and primitive reflexes. Respiratory rate was 50 breaths per minute, saturation was well-maintained at 98%, and Silverman's score was 2/10. Hemodynamically, the newborn was tachycardic at 186 beats per minute with a capillary refill time of less than three seconds. There was a hyperdynamic precordium with continuous cardiac murmur on cardiac auscultation. Abdominal examination revealed hepatomegaly with a liver span of 6 cm. Malformation screening was negative.

**Figure 1:**
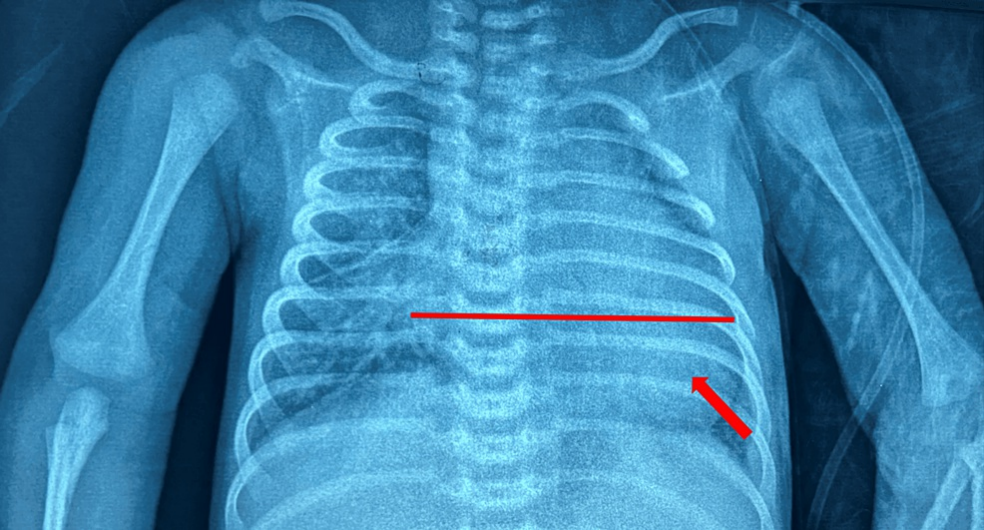
Front thoracic X-ray showing cardiomegaly at 0.65 with a convex left lower arch.

**Figure 2:**
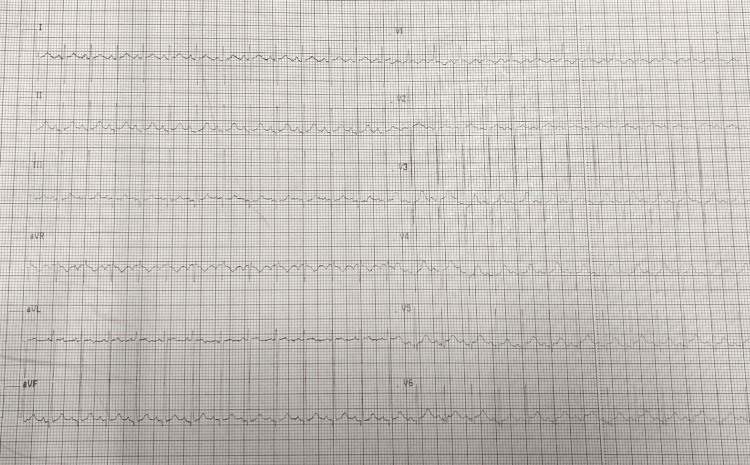
Electrocardiogram.

A chest radiograph (Figure 1) was initially performed, showing cardiomegaly with a cardiothoracic ratio of 0.65 and convexity of the left lower arch. The electrocardiogram was normal (Figure 2).

**Figure 3:**
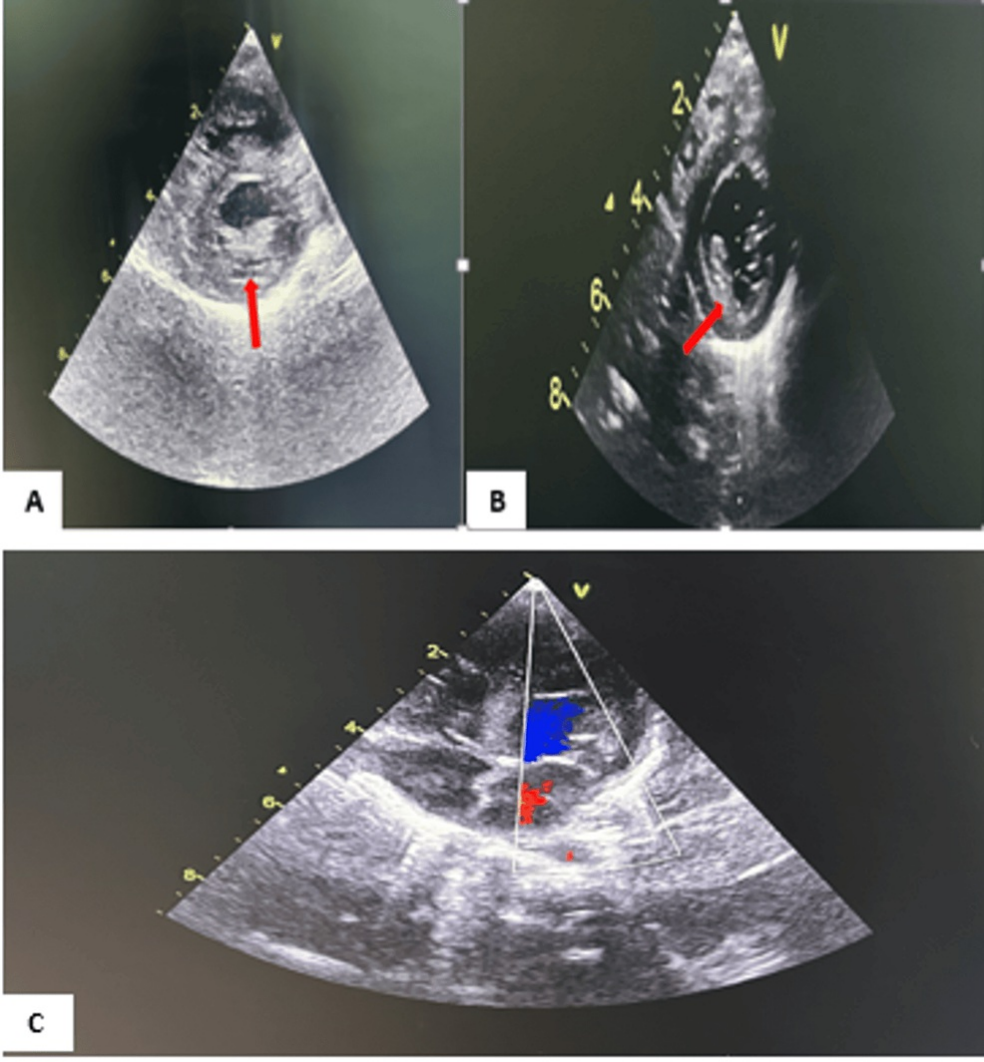
Echocardiographic image of the patient at admission. A: Short-axis parasternal section showing left ventricular hypertrophy with ejection fraction at 43%; B: short-axis parasternal section showing a 6 mm thickening of the posterior wall; C: four-chamber color Doppler imaging showing the overall cardiac morphology of our patient

Echocardiography (Figure 3) showed left ventricular hypertrophy, predominantly septal, with a thickened posterior wall measuring 6 mm, hypokinetic with an ejection fraction of 43%. All parameters measured by echocardiography that led to the diagnosis are presented in Table 1.

**Table 1:** Different echocardiographic parameters studied and their interpretations based on the Z-score.

Parameter	Found value	Reference value	Z-score	Severity classification
Interventricular septum (IVS) thickness (mm)	7 mm	3-6 mm (newborn)	>3	Moderate to severe hypertrophy
Posterior wall of the left ventricle (mm)	6 mm	3-6 mm (newborn)	>2	Normal to slightly hypertrophic
Left ventricular end-diastolic diameter (mm)	24 mm	13-17 mm (newborn)	>3	Ventricular dilation

The pro-brain natriuretic peptide (Pro-BNP) was elevated at 14,315 pg/mL. The newborn was started on oral furosemide at a dose of 2 mg/kg/day with potassium supplementation, which led to significant improvement. Three days after starting treatment, the newborn became normocardic at 135 beats per minute, hepatomegaly decreased over time, and the baby regained autonomy in feeding and breathing.

**Figure 4:**
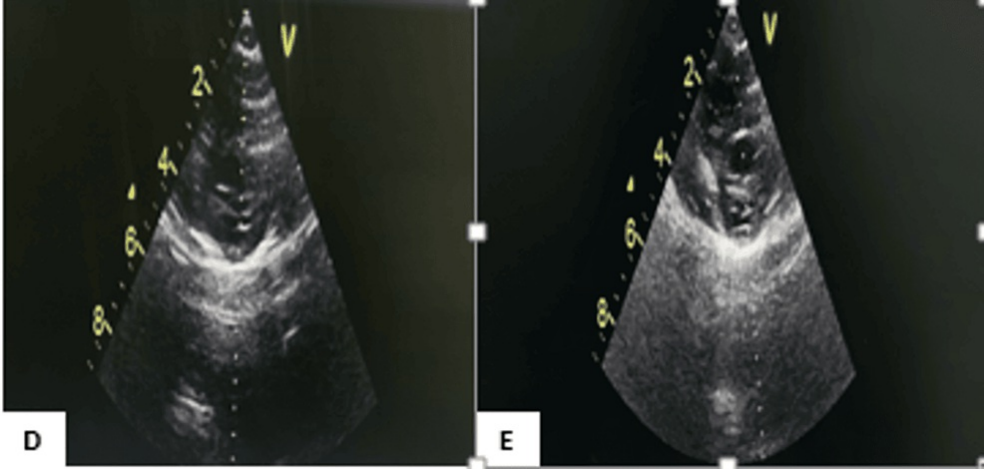
Cardiac ultrasound image of our patient showing initial improvement. D: Short-axis parasternal section showing posterior wall thickness at 4 mm; E: short-axis parasternal section showing left ventricular hypertrophy with ejection fraction at 66%

A follow-up echocardiogram performed 15 days later showed an improvement in ejection fraction to 66% and a reduction in posterior wall thickness to 4 mm. Echocardiography was repeated at three months and showed normalization (Figure 4).

On an etiological level, a series of biological tests were conducted including the following: L-carnitine levels were normal at 31 μmol/L. In the search for a metabolic disorder, lactate, ammonia, and lactate dehydrogenase (LDH) levels were normal (Table 2). A Guthrie test was performed and was normal. Genetic testing was conducted and revealed no abnormalities: no mutations responsible for myopathy were detected. The karyotype was normal. Ultimately, following this series of tests and the rapid regression of symptoms, the diagnosis of hypokinetic HCM in response to prenatal betamethasone exposure was confirmed.

**Table 2:** Biological values of our patient. SGOT: serum glutamic oxaloacetic transaminase; SGPT: serum glutamic pyruvic transaminase; Pro-BNP: Pro-brain natriuretic peptide; U/L: units per liter

Laboratory parameters	Initial values	Reference ranges
Hemoglobin (g/dL)	13.9	16.5-18.5
SGOT (U/L)	36	20-80
SGPT (U/L)	17	2-20
Pro-BNP (Pg/mL)	14,315	<3,000
Lactate dehydrogenase (U/L)	664	500-1,200
Lactate(mmol/L)	1.5	1-1.65
Ammonium (Umol/L)	51	37-63
L-carnitine (Umol/L)	31	20-50
Troponin (ng/mL)	0.03	<0.05

Regular echocardiographic follow-up showed complete normalization of the ejection fraction after three months.

## Discussion

Neonatal HCM is an exceptional entity in neonates [2,5]. It is a primary disorder of the cardiac muscle marked by an enlarged but non-dilated left ventricle, associated or not with a systolic intraventricular pressure gradient [6]. The annual incidence of this disease is 1.24 per 100,000 children under the age of 10. It is responsible for 10% of pediatric cardiac deaths [7]. Histologically, this condition is characterized by myocyte disorganization. Moreover, it should be remembered that 60% of patients described in the literature had a family history, with several siblings affected, explaining the high degree of chromosomal dominance, which explains the genetic origin frequently found because of missense mutations in the genes coding for sarcomeric proteins [2,7]. The absence of a family history or genetic mutations in the patient suggests potentially acquired or sporadic causes [5]. Hence, carefully exploring environmental factors and intra-uterine conditions is important.

Clinical manifestations in newborns and infants are highly atypical: in our patient, the simultaneous presence of symptoms such as neonatal respiratory distress, fatigue during suckling, and physical signs such as hepatomegaly and tachycardia suggest a possible underlying cardiovascular pathology. Age at presentation is a determining factor in prognosis [1]. The clinical picture in newborns is mainly one of congestive heart failure, as in our case, which can progress to serious complications, including sudden death [1,7].

Cardiac ultrasonography is a key diagnostic tool, enabling the diagnosis, intervention, follow-up, and estimation of the risk of sudden death [2,5,7]. Chest X-rays are often normal in the neonatal period and the first few months of life and may show cardiomegaly in cases of associated heart failure, as in our patient's case. The abnormalities detected by electrocardiography can help in the diagnosis and are present in over 75% of cases, revealing left atrial hypertrophy, left ventricular hypertrophy, Q wave, R wave, or ST segment abnormalities [4,5]. Our patient, on the other hand, had no abnormalities found on ECG. At present, MRI is finding its place more and more, providing additional information compared with echocardiography. However, the constraint is its high cost and the risks associated with sedation [4].

The literature reports that around 69.2% of HCM affecting newborns and infants before the age of one is considered idiopathic [1]. HCM is classified as either obstructive or nonobstructive. Whether the obstruction is present or absent, it may be familial in origin: sarcomeric, or linked to syndromes such as Noonan or Friedreich's ataxia, as well as appearing in the context of certain metabolic disorders or myopathies [1]. Iatrogenic nonfamilial forms are seen in newborns of diabetic mothers or after the administration of certain drugs such as corticosteroids. Indeed, several papers have been written on the relationship between corticosteroid treatment for the prevention of bronchoalveolar dysplasia in premature infants and the occurrence of HCM, with reversible dose-dependent changes immediately after discontinuation of the drug [8,9]. However, cases secondary to antenatal corticosteroid injections are very rare and limited to a few trials [5,6,10]. Studies suggest that corticosteroids given to pregnant women at risk of preterm delivery may increase cardiac output and blood pressure as well as the occurrence of transient HCM in newborns whose mothers had received repeated doses of antenatal corticosteroids [6,10]. This hypothesis remains the most likely explanation for the occurrence of HCM.

Therapeutic management is symptomatic, aimed at improving the patient's quality of life, and relies first and foremost on beta-blockers [1]. Calcium channel blockers (verapamil) are used in intolerant patients. Furosemide (Lasilix) is indicated for short periods and patients with congestive heart failure. Heart transplantation is indicated for end-stage HCM. The prognosis for newborns and infants is worse than for older children. Age is an important prognostic factor [11], and they can rapidly progress to congestive heart failure. Our patient progressed well under symptomatic treatment, which may be explained by the rapidity of diagnosis and management.

## Conclusions

In our context, corticosteroid-induced HCM in newborns is underrecognized, often leading to significant diagnostic delays. Emphasizing the uniqueness of our observation is crucial, as our case represents one of the first documented presentations of this condition in Morocco. Despite several studies exploring the postnatal use of corticosteroids in the context of bronchopulmonary dysplasia and HCM, this association remains rare. The limited incidence of this condition linked to prenatal corticosteroid exposure underscores its significance in the medical literature.

We aim to raise awareness among practitioners, especially pediatricians, about the potential risk of developing HCM in newborns exposed to prenatal corticosteroids. Adequate awareness could facilitate early and tailored management, thereby improving clinical outcomes for these vulnerable patients. This rare case of hypokinetic HCM in a newborn highlights the complex challenges associated with managing cardiac disorders in infants.
